# Induction of labor with Foley catheter and risk of subsequent preterm birth: follow‐up study of two randomized controlled trials (PROBAAT‐1 and ‐2)

**DOI:** 10.1002/uog.23117

**Published:** 2021-02-01

**Authors:** M. D. T. de Vaan, D. Blel, K. W. M. Bloemenkamp, M. Jozwiak, M. L. G. ten Eikelder, J. W. de Leeuw, M. A. Oudijk, J. J. H. Bakker, R. J. P. Rijnders, D. N. Papatsonis, M. Woiski, B. W. Mol, R. de Heus

**Affiliations:** ^1^ Department of Obstetrics and Gynaecology Jeroen Bosch Hospital ‘s‐Hertogenbosch The Netherlands; ^2^ Department of Health Care Studies Rotterdam University of Applied Sciences Rotterdam The Netherlands; ^3^ Department of Obstetrics and Gynaecology Ikazia Hospital Rotterdam The Netherlands; ^4^ Department of Obstetrics, Division Woman and Baby, Wilhelmina Children's Hospital Birth Centre University Medical Centre Utrecht Utrecht The Netherlands; ^5^ Department of Gynaecologic Oncology Erasmus Medical Centre Rotterdam The Netherlands; ^6^ Department of Obstetrics and Gynaecology, Princess Alexandra Wing Royal Cornwall Hospital NHS Trust Truro UK; ^7^ Department of Obstetrics Amsterdam Reproduction and Development Research Institute, Amsterdam UMC, University of Amsterdam Amsterdam The Netherlands; ^8^ Department of Obstetrics and Gynaecology Amphia Hospital Breda The Netherlands; ^9^ Department of Obstetrics and Gynaecology Radboud University Medical Centre Nijmegen The Netherlands; ^10^ Department of Obstetrics and Gynaecology Monash University Melbourne Australia; ^11^ Aberdeen Centre for Women's Health Research University of Aberdeen Aberdeen UK

**Keywords:** balloon, cervical ripening, Foley catheter, induction of labor, preterm birth

## Abstract

**Objective:**

To evaluate the rate of preterm birth (PTB) in a subsequent pregnancy in women who had undergone term induction using a Foley catheter compared with prostaglandins.

**Methods:**

This was a follow‐up study of two large randomized controlled trials (PROBAAT‐1 and PROBAAT‐2). In the original trials, women with a term singleton pregnancy with the fetus in cephalic presentation and with an indication for labor induction were randomized to receive either a 30‐mL Foley catheter or prostaglandins (vaginal prostaglandin E2 in PROBAAT‐1 and oral misoprostol in PROBAAT‐2). Data on subsequent ongoing pregnancies > 16 weeks’ gestation were collected from hospital charts from clinics participating in this follow‐up study. The main outcome measure was preterm birth < 37 weeks’ gestation in a subsequent pregnancy.

**Results:**

Fourteen hospitals agreed to participate in this follow‐up study. Of the 1142 eligible women, 572 had been allocated to induction of labor using a Foley catheter and 570 to induction of labor using prostaglandins. Of these, 162 (14%) were lost to follow‐up. In total, 251 and 258 women had a known subsequent pregnancy > 16 weeks' gestation in the Foley catheter and prostaglandin groups, respectively. There were no differences in baseline characteristics between the groups. The overall rate of PTB in a subsequent pregnancy was 9/251 (3.6%) in the Foley catheter group *vs* 10/258 (3.9%) in the prostaglandin group (relative risk (RR), 0.93; 95% CI, 0.38–2.24), and the rate of spontaneous PTB was 5/251 (2.0%) *vs* 5/258 (1.9%) (RR, 1.03; 95% CI, 0.30–3.51).

**Conclusion:**

In women with term singleton pregnancy, induction of labor using a 30‐mL Foley catheter is not associated with an increased risk of PTB in a subsequent pregnancy, as compared to induction of labor using prostaglandins. © 2020 The Authors. Ultrasound in Obstetrics & Gynecology published by John Wiley & Sons Ltd on behalf of International Society of Ultrasound in Obstetrics and Gynecology.

**CONTRIBUTION**

**What are the novel findings of this work?**

In women with an unripe cervix scheduled for induction of labor, induction using a Foley catheter does not seem to increase the risk of preterm birth in a subsequent pregnancy, as compared with induction using prostaglandins.

**What are the clinical implications of this work?**

Clinicians should not be hesitant in using a Foley catheter for induction of labor based on the hypothetical increased risk of preterm birth in a subsequent pregnancy, as we did not find such an association and as it has a more optimal neonatal safety profile compared to pharmacological methods.

## INTRODUCTION

Labor induction is a common obstetric procedure, which is generally carried out when the risk of continuing pregnancy outweighs the benefit. In the USA, approximately one in four women is induced and, in the last decade, the induction rate in the UK has risen to almost 30%^1^, ^2^.

Mechanical induction using a Foley catheter has gained popularity as it has a better safety profile compared to the conventionally used dinoprostone, reducing the risk of neonatal morbidity while being equally effective^3^, ^4^. However, there is a hypothetical risk that the mechanical stretch of the cervix by the balloon can cause damage to the cervical tissue and, as a result, may affect the risk of preterm birth (PTB) in a subsequent pregnancy.

Cervical abnormalities, either congenital or as a result of trauma, are a risk factor for structural cervical weakness, which can lead to PTB. Known traumas associated with spontaneous PTB are mechanical cervical dilation during a gynecologic procedure and treatment of cervical intraepithelial neoplasia^5^, ^6^. It is unknown whether a balloon catheter for labor induction has a similar traumatizing effect on cervical integrity, which could lead to spontaneous PTB in a subsequent pregnancy. Studies examining the association between induction of labor using a balloon catheter and subsequent PTB are few and provide low‐quality evidence, and no data from randomized controlled trials (RCTs) are available to answer the question of whether a balloon catheter increases the risk of PTB in a subsequent pregnancy^7^, ^8^, ^9^.

Our previous PROBAAT trials, two large multicenter RCTs, showed that cervical ripening using a Foley catheter reduced the risk of fetal distress, as compared to cervical ripening using prostaglandin E2, whereas there was no difference in outcome when compared to cervical ripening using oral misoprostol. In both studies, no difference was seen in Cesarean section rate between the arms^3^, ^10^.

The aim of this study was to evaluate the rate of PTB in a subsequent pregnancy in women who had undergone term induction using a Foley catheter compared to prostaglandins, in order to assess the long‐term safety outcome of Foley catheter induction, by performing a follow‐up study of two RCTs.

## METHODS

We designed a follow‐up study for the PROBAAT‐1 and PROBAAT‐2 trials. Both studies were multicenter RCTs, for which the full methods and results have been published elsewhere^3^, ^10^. In brief, the PROBAAT‐1 trial randomized 819 women, between February 2009 and May 2010, to induction of labor using a 30‐mL Foley catheter (*n* = 411) or vaginal prostaglandin E2 gel (*n* = 408). The PROBAAT‐2 trial randomized 1845 women, between July 2012 and October 2013, to induction of labor using a 30‐mL Foley catheter (*n* = 921) or oral misoprostol (*n* = 924).

In total, 27 hospitals collaborating in the Dutch Consortium for Healthcare Evaluation and Research in Obstetrics and Gynaecology (NVOG Consortium 2.0) participated in one or both of the PROBAAT trials. Both trials were approved by the Central Committee on Research Involving Human Subjects, by the ethics committee of the Academic Medical Center, Amsterdam, The Netherlands and by the board of directors of each participating hospital and were registered with the Dutch Trial Registry (NTR 1646 and NTR3466). The ethics committee judged that the Research Involving Human Subjects Act did not apply to this retrospective follow‐up study and therefore no further approval was required (date of approval 15 March 2018, ref. W18‐098 #18.024). As data collection for this follow‐up study was not prespecified in the original trial protocols, additional approval of data collection for the purpose of this follow‐up study was obtained from the board of directors of each participating hospital.

Both PROBAAT trials included pregnant women scheduled for induction of labor beyond 37 weeks of gestation with a live singleton pregnancy with the fetus in cephalic presentation, intact membranes and an unfavorable cervix (Bishop score < 6). Women younger than 18 years of age and those with a previous Cesarean section, placenta previa, lethal fetal congenital anomaly or known hypersensitivity to one of the products used for induction were ineligible.

The pregnancy at the time of the PROBAAT trials was considered the index pregnancy. For this follow‐up study, we included only women who had a subsequent ongoing pregnancy beyond 16 weeks' gestation after participation in one of the PROBAAT trials. In the subsequent pregnancy, gestational age was determined by first‐trimester measurement of crown–rump length. No routine cervical length screening was performed, and progesterone was administered only when indicated according to local protocol. No further exclusion criteria were specified.

Details on randomization and interventions for each trial have been described previously^3^, ^10^. In short, after written informed consent was provided, women were allocated randomly to induction of labor using either a Foley catheter or prostaglandins by their attending physician, in a 1:1 ratio, using an online program.

**Figure 1:**
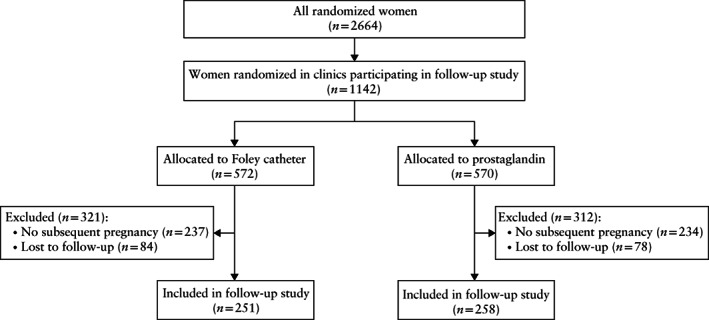
Flowchart summarizing inclusion in follow‐up study of women randomized to induction of labor using Foley catheter or prostaglandin, who had subsequent pregnancy.

In both studies, women allocated to induction using a Foley catheter had a 16‐Fr or 18‐Fr Foley catheter introduced through the cervix, either digitally or using a vaginal speculum. After the Foley catheter had passed the internal ostium, the balloon was filled with 30 mL of 0.9% saline or sterile water. The external end of the Foley catheter was taped to the inner thigh without traction. If the Bishop score remained < 6 after 24 h, the location of the Foley catheter was checked. When still in correct position, the Foley catheter was either left in place (PROBAAT‐2) or replaced with a new one after 24 h (PROBAAT‐1). If the Bishop score remained < 6 after 48 h, the catheter was replaced.

Women allocated to prostaglandin E2 (in PROBAAT‐1) were treated mostly with a starting dose of 1 mg prostaglandin E2 gel, followed by 1 mg after 6 h, with a maximum of two doses per 24 h, inserted into the posterior vaginal fornix. An initial dose of 2 mg was allowed in nulliparous women, as prescribed by the manufacturer (Pfizer, New York, NY, USA). Women allocated to oral misoprostol (in PROBAAT‐2) received 50 µg capsules once every 4 h for a maximum of three times daily.

In both trials, if the cervix was still unfavorable for amniotomy after 48 h of treatment, women were generally assigned a day of rest followed by another 48 h of induction.

For this follow‐up study, the databases of both studies were combined, and study allocation was regrouped into Foley catheter or prostaglandins. To assess eligibility, electronic hospital records of women who participated in one of the PROBAAT trials were searched manually by the first and second authors. If a subsequent ongoing pregnancy after the index pregnancy had occurred, the required data for this study were collected. If it was not clear if there had been a subsequent pregnancy, the case was classified as lost to follow‐up. For women who had more than one subsequent, ongoing pregnancy, only the first was included. Missing data are noted in the tables. To eliminate the potential bias of an unknown history of mechanical dilatation before a surgical abortion or PTB, we also performed an analysis limited to women who had two consecutive pregnancies and were primigravid during the index pregnancy.

The main outcome of this follow‐up study was PTB < 37 weeks' gestation in a subsequent pregnancy. Other outcomes were spontaneous PTB < 34 and < 37 weeks' gestation, gestational age at delivery, type of onset of labor, mode of delivery and birth weight in a subsequent pregnancy. For the outcome spontaneous PTB in a subsequent pregnancy, women with iatrogenic onset of labor (i.e. induction of labor or planned Cesarean section) or multiple pregnancy were excluded.

Data were analyzed on an intention‐to‐treat basis. Numerical variables were summarized as mean ± SD and analyzed using the *t‐*test if the distribution was normal. When the distribution was skewed, they were summarized as median with interquartile range and analyzed using Mann–Whitney *U*‐test. The χ^2^ test or Fisher's exact test was used to compare categorical variables. Treatment effect was presented as relative risk (RR) with 95% CI. A *P‐*value of < 0.05 was considered to indicate statistical significance. Statistical analyses were performed using SPSS version 21.0 (IBM Corp., Armonk, NY, USA).

## RESULTS

Of the 27 hospitals that participated in the PROBAAT trials, 14 agreed to participate in this follow‐up study. The main reason for hospitals not participating in this follow‐up study was the time‐consuming process with no funding available to compensate the hospitals for their work. Data were collected between January 2018 and November 2018.

During the original trial periods, 2664 women were randomized in the PROBAAT trials, of whom 1142 were randomized in the 14 hospitals participating in the current study (Figure 1). The baseline characteristics of the women from clinics that participated in this follow‐up study were comparable to those from clinics that did not participate, except for parity, the rate of which was slightly higher in women in the participating clinics (Table S1).

Of the 1142 women randomized in the 14 participating hospitals, 572 had been allocated to induction of labor using a Foley catheter and 570 to induction of labor using prostaglandins. Of these women, 237 (41%) and 234 (41%) did not have a subsequent, ongoing pregnancy, and 84 (15%) and 78 (14%) were lost to follow‐up, respectively. We therefore included 251 women in the Foley catheter group and 258 women in the prostaglandin group (Figure 1).

Baseline characteristics of the women with a subsequent pregnancy after the PROBAAT studies are shown in Table 1. The groups were comparable with respect to age, body mass index at booking, ethnicity, parity and mode of delivery of the index pregnancy. For women who had an ongoing pregnancy prior to the index pregnancy, no information was available on whether they had a previous PTB. In the Foley catheter group, six women had a multiple gestation in the subsequent pregnancy, compared with five in the prostaglandin group. There was no record of any of the included women receiving progesterone for prevention of PTB in the subsequent pregnancy.

**Table 1:** Baseline characteristics of index pregnancy in 509 women who underwent induction of labor and had subsequent pregnancy, according to randomization to Foley catheter or prostaglandins

Characteristic	Foley catheter (*n* = 251)	Prostaglandin (*n* = 258)	*P*
Parity			0.465§
Nulliparous	204 (81)	203 (79)	
Parous	47 (19)	55 (21)	
Body mass index (kg/m^2^)	25 (22–28)†	26 (22–29)‡	0.849¶
Ethnic origin*			0.858§
Caucasian	202/241 (84)	208/252 (83)	
Non‐Caucasian	40/241 (17)	43/252 (17)	
Maternal age (years)	29 ± 4.5	30 ± 4.4	0.052**
Mode of delivery			0.181§
Spontaneous	164 (65)	172 (67)	
Assisted vaginal	28 (11)	42 (16)	
Cesarean section	59 (24)	44 (17)	

Data are given as *n* (%), median (interquartile range), *n*/*N* (%) or mean ± SD. Data missing for:

* 16 cases;

† 26 cases;

‡ 34 cases.

§ Chi‐square test.

¶ Mann–Whitney *U*‐test.

** *t*‐test.

No difference was found in the rate of PTB < 37 weeks' gestation in the subsequent pregnancy between the Foley catheter and prostaglandin groups (9/251 (3.6%) *vs* 10/258 (3.9%); RR, 0.93; 95% CI, 0.38–2.24). After excluding women with iatrogenic PTB and those with multiple pregnancy, the rate of PTB < 37 weeks in the subsequent pregnancy remained comparable between the groups, occurring in five women per group (2.0% *vs* 1.9%; RR, 1.03; 95% CI, 0.30–3.51). In the Foley catheter group, no woman had subsequent spontaneous PTB < 34 weeks' gestation, compared with two women in the prostaglandin group. No differences between the groups were found in gestational age at delivery, type of onset of labor, mode of delivery or neonatal birth weight in the subsequent pregnancy (Table 2).

**Table 2:** Obstetric outcomes of subsequent pregnancy in 509 women who underwent induction of labor in index pregnancy, according to randomization to Foley catheter or prostaglandins

Outcome	Foley catheter (*n* = 251)	Prostaglandin (*n* = 258)	RR (95% CI)	*P*
All PTB < 37 weeks	9 (3.6)	10 (3.9)	0.93 (0.38–2.24)	0.864
Spontaneous PTB < 37 weeks*	5 (2.0)	5 (1.9)	1.03 (0.30–3.51)	0.965
Spontaneous PTB < 34 weeks*	0 (0)	2 (1.0)	NA	0.256**
GA at delivery (weeks)	39 + 5 (38 + 5 to 41 + 0)§	39 + 4 (38 + 2 to 40 + 5)¶	NA	0.068††
Multiple pregnancy	6 (2.4)	5 (1.9)	1.23 (0.38–3.99)	0.726
Onset of labor†				
Spontaneous	105/199 (52.8)	129/226 (57.1)	0.93 (0.78–1.10)	0.372
Induction	68/199 (34.2)	75/226 (33.2)	1.03 (0.79–1.35)	0.830
Elective Cesarean section	26/199 (13.1)	22/226 (9.7)	1.34 (0.77–2.29)	0.279
Mode of delivery‡				
Spontaneous	190/242 (78.5)	199/244 (81.6)	0.96 (0.88–1.05)	0.401
Assisted vaginal	8/242 (3.3)	5/244 (2.0)	1.61 (0.54–4.86)	0.391
Cesarean section	44/242 (18.2)	40/244 (16.4)	1.11 (0.75–1.64)	0.602
Birth weight (g)	3566 ± 544†	3530 ± 511‡	NA	0.501‡‡

Data are given as *n* (%), median (interquartile range), *n*/*N* (%) or mean ± SD.

* Including only preterm births (PTB) in singleton pregnancies with non‐iatrogenic onset of labor. Data missing for:

† 84 cases (52 in Foley group and 32 in prostaglandin group);

‡ 23 cases (nine in Foley group and 14 in prostaglandin group);

§ 33 cases;

¶ 52 cases.

*P*‐values calculated using chi‐square test unless stated otherwise.

** Fisher's exact test.

†† Mann–Whitney *U*‐test.

‡‡ *t*‐test.

GA, gestational age; NA, not applicable; RR, relative risk.

Per protocol analysis of women who actually received their allocated treatment method did not affect the results. Eleven women allocated to Foley catheter were induced with prostaglandin, mainly because of failed placement. Four women allocated to prostaglandin received a Foley catheter at some point during the induction process. None of the women who had crossover of induction methods had PTB in the subsequent pregnancy. *Post‐hoc* analysis of women with two consecutive singleton pregnancies and who were primigravid during the index pregnancy, showed no difference in the rate of PTB < 37 weeks in the second pregnancy between the Foley catheter and prostaglandin groups (4/172 (2.3%) *vs* 2/143 (1.4%); RR, 1.66; 95% CI, 0.31–8.95). Analyzing separately oral misoprostol and prostaglandin E2 *vs* Foley catheter had no effect on the observed outcome.

## DISCUSSION

### Main findings

The findings of this follow‐up study of two RCTs comparing mechanical induction of labor using a Foley catheter to induction using prostaglandins, have shown that Foley catheter is not associated with an increased risk of PTB in the subsequent pregnancy.

### Strengths and limitations

Our follow‐up data were based on two large RCTs, which minimized the risk of bias for other, sometimes unknown, confounding factors. In these RCTs, baseline characteristics were similar between the groups, and the methods of induction were well‐defined. Also, the fact that, in the control arms of both studies, women were induced by pharmacological methods, rather than having spontaneous onset of labor, allows comparison to a relevant control group, as the findings of some studies have suggested that spontaneous term birth up to 39 weeks' gestation could be associated with subsequent PTB^8^, ^11^. However, some undetected bias could still be present. For instance, no information was available on whether mechanical cervical dilation during a gynecologic procedure or treatment of cervical intraepithelial neoplasia was performed in the period between the index pregnancy and subsequent pregnancy^5^, ^6^. Also, no information was available on some other known factors associated with an increased risk of PTB, such as the interval between the index pregnancy and subsequent pregnancy, the incidence of PTB in a previous pregnancy before the index pregnancy or socioeconomic factors^12^, ^13^. Despite the possible presence of confounders, the study design makes it likely that, if present, they are equally distributed between both groups.

In the 14 participating hospitals with 1142 included patients, of whom 509 had a known subsequent pregnancy, the loss‐to‐follow‐up rate was 14%, which is generally accepted^14^. Since we only had access to hospital records, women with a low‐risk subsequent pregnancy who had only midwife‐led care might have been missed. However, the PTB rate in the lost to follow‐up group is likely to be zero, because women with preterm labor are usually referred to the same hospital (regional referral system).

While we included 509 women, our study could be underpowered to detect a clinically relevant difference in the rate of PTB between the groups. For example, given the 3.9% rate of PTB < 37 weeks' gestation found in the prostaglandin group, a sample size of 1134 women would be required to detect a two‐fold increase in PTB (power, 80%; alpha error, 5%). Moreover, the sample size had to be even higher for more clinically relevant cut‐off points, such as PTB < 34 or < 32 weeks. However, this study did not show any trend towards a difference in the rate of PTB between the groups, and the *a‐priori* risk of PTB in our population is very low.

### Interpretation

Our findings are in line with those of other studies addressing the same research question^7^, ^8^, ^9^. Zafran and colleagues^9^ compared, in their cohort study of 366 women with two or more known pregnancies, term induction of labor using a balloon catheter (60‐mL Foley catheter or Cook double balloon) with term induction using vaginal prostaglandin E2, as well as with spontaneous onset of labor and found no difference in the rate of spontaneous PTB < 37 weeks' gestation in a subsequent pregnancy (0.8% *vs* 0.9% *vs* 3.1%; *P* = 0.38). Sciscione and colleagues^7^ compared, in their cohort study of 126 women with two or more known pregnancies, term induction of labor using a 30‐mL Foley catheter with applied traction to term induction using vaginal prostaglandin E2, and also found no difference in the rate of PTB < 37 weeks' gestation in a subsequent pregnancy (3.2% *vs* 4.7%; *P =* 0.53). Levine and colleagues^8^ also found no association between induction of labor using a Foley catheter, as compared with spontaneous onset of labor, and PTB in a subsequent pregnancy in their cohort of 887 women (adjusted odds ratio, 0.41; 95% CI, 0.15–1.12).

Although all of the above studies addressed the same research question, heterogeneity exists between studies with regard to the balloon volume and whether or not traction was applied. In our study, a 30‐mL Foley catheter was placed above the internal ostium and used without traction. Looking at contributing factors for cervical weakness, it is reasonable to believe that using a larger balloon volume or a double Cook balloon, in which an 80‐mL balloon is placed above and below the internal ostium, or applying traction, could have the potential to cause more cervical tissue damage and therefore have more effect on cervical integrity. However, such an association was not found by researchers who used these methods^7^, ^9^. High‐volume balloons, double balloons or applying traction are used to expedite labor. However, not all of these strategies have been proven to shorten this process. Although a double balloon or applying traction may shorten the period from start of induction to expulsion of the balloon, these approaches do not increase the number of vaginal deliveries within 24 h and have the disadvantage of causing more pain during the induction process^15^, ^16^, ^17^, ^18^.

In the present follow‐up study, the PTB rate was 3.7%, which is low in comparison to the national PTB rate of 7.2% in The Netherlands^19^. This could be explained by the fact that women in this study had induction of labor at term in their index pregnancy and had a relatively low‐risk pregnancy, which could influence the baseline risk of PTB in this specific population. This hypothesis is also supported by the comparable numbers of PTB in relatively similar populations. Baer and colleagues^20^, in a Californian cohort of 133 662 women, found a PTB rate of 3.2% after a previous term birth between 39 and 42 weeks of gestation. Furthermore, Zafran *et al*.^9^ and Sciscione and colleagues^7^ found relatively low numbers of PTB in the subsequent pregnancy after term induction using a Foley catheter (0.5% (spontaneous PTB) and 3.2%, respectively). Levine and colleagues^8^ are the only ones to report a PTB rate of 10% in a subsequent pregnancy after term labor induction. However, the spontaneous PTB rate was 6%, while in women induced with a Foley catheter, the spontaneous PTB rate was 4%.

### Conclusion

In women with an unripe cervix scheduled for induction of labor, induction using a Foley catheter does not seem to increase the risk of PTB in a subsequent pregnancy, as compared with induction using prostaglandins.

## Supporting information


**Table S1** Baseline characteristics of women randomized to induction of labor using Foley catheter or prostaglandin, according to whether they were randomized in clinic that participated in follow‐up study
