# Supplementary material for: Induction of labor with Foley catheter and risk of subsequent preterm birth: follow‐up study of two randomized controlled trials (PROBAAT‐1 and ‐2)
Source: Ultrasound Obstet Gynecol. 2021 Feb 1;57(2):292–7. doi: 10.1002/uog.23117 (PMC7898639; doi:10.1002/uog.23117)
Supplement: Supplementary file 1 — Table S1 Baseline characteristics of women randomized to induction of labor using Foley catheter or prostaglandin, according to whether they were randomized in clinic that participated in follow‐up study [file UOG-57-292-s001.doc]

| **Table S1** Baseline characteristics of women randomized to induction of labor using Foley catheter or prostaglandin, according to whether they were randomized in clinic that participated in follow-up study | | | | |
| --- | --- | --- | --- | --- |
|  | **Participating clinics**  **n=1142** | **Non-participating clinics**  **n=1522** | | ***p*-value** |
| **Parity**  **nulliparous**  **multiparous** | 708 (62%)  434 (38%) | 1029 (68%)  493 (32%) | | 0.003 |
| **Body Mass Index** | 25^1^ [22-29] | 25^2^ [22-29] | | 0.907† |
| **Ethnic origin^3^**  **Caucasian**  **Non-Caucasian** | 911 (84%)  179 (17%) | 1169 (84%)  236 (17%) | 0.803 | |
| **Maternal age (years)** | 31 (±5.0) | 31 (±5.1) | 0.903‡ | |
| **Mode of delivery in PROBAAT-trial**  **Spontaneous delivery**  **Assisted vaginal delivery**  **Caesarean section** | 807 (71%)  120 (10%)  215 (19%) | 1030 (68%)  192 (12%)  300 (20%) | 0.167 | |
| **Values are given as numbers, (%), mean (±SD) or median [IQR]. † Mann-Whitney-U test ‡ t-test**  **^1^ 154 cases missing**  **^2^ 104 cases missing**  **^3^ 169 cases missing** | | | | |
